# DFT-Based Functionalization of Graphene with Lithium-Modified Groups for Enhanced Hydrogen Detection: Thermodynamic, Electronic, and Spectroscopic Properties

**DOI:** 10.3390/nano15161234

**Published:** 2025-08-13

**Authors:** Norma A. Rangel-Vázquez, Adrián Bonilla-Petriciolet, Edgar A. Márquez-Brazón, Yectli Huerta, Rosa Zavala-Arce, Juan D. Rodríguez-Macías

**Affiliations:** 1 TecNM/Instituto Tecnológico de Aguascalientes, Avenida Adolfo López Mateos 1801, Aguascalientes 20256, Mexico; adrian.bp@aguascalientes.tecnm.mx; 2 Grupo de Investigaciones en Química y Biología, Departamento de Química y Biología, Facultad de Ciencias Básicas, Universidad del Norte, Carrera 51B, Km 5, Vía Puerto Colombia, Barranquilla 081007, Colombia; 3 Minnesota Supercomputing Institute, University of Minnesota, Minneapolis, MN 55455, USA; yhuerta@umn.edu; 4 TecNM/Instituto Tecnológico de Toluca, Avenida Tecnológico 100 s/n, Metepec 52149, Mexico; rzavalaa@toluca.tecnm.mx; 5 Facultad de Ciencias de la Salud, Exactas y Naturales, Universidad Libre, Barranquilla 080001, Colombia; juand.rodriguezm@unilibre.edu.co

**Keywords:** DFT simulation, FTIR, electronic distribution, hydrogen, thermodynamic properties, spectroscopic properties

## Abstract

This study investigates the impact of oxygen-containing functional groups (COO-Li, CO-Li, and O-Li) on the electronic and optical properties of graphene, with a focus on hydrogen sensing applications. Using density functional theory (DFT) calculations, we evaluated the thermodynamic feasibility of the functionalization and hydrogen adsorption processes. The Gibbs free energy changes (ΔG) for the functionalization of pristine graphene were calculated as −1233, −1157, and −1119 atomic units (a.u.) for COO-Li, CO-Li, and O-Li, respectively. These negative values indicate that the functionalization processes are spontaneous (ΔG < 0), with COO-Li being the most thermodynamically favorable. Furthermore, hydrogen adsorption on the functionalized graphene surfaces also exhibited spontaneous behavior, with ΔG values of −1269, −1204, and −1175 a.u., respectively. These results confirm that both functionalization and subsequent hydrogen adsorption are energetically favorable, enhancing the potential of these materials for hydrogen sensing applications. Among the functional groups we simulated, COO-Li exhibited the largest surface area and volume, which were attributed to the high electronegativity and steric influence of the carboxylate moiety. Based on the previously described results, we analyzed the interaction of these functionalized graphene systems with molecular hydrogen. The adsorption of two H_2_ molecules per system demonstrated favorable thermodynamics, with lithium atoms serving as active sites for external adsorption. The presence of lithium atoms significantly enhanced hydrogen affinity, suggesting strong potential for sensing applications. Further, electronic structure analysis revealed that all functionalized systems exhibit semiconducting behavior, with band gap values modulated by the nature of the functional group. FTIR (Fourier-Transform Infrared Spectroscopy) and Raman spectroscopy confirmed the presence of characteristic vibrational modes associated with Li-H interactions, particularly in the 659–500 cm^−1^ range. These findings underscore the promise of lithium-functionalized graphene, especially with COO-Li, as a tunable platform for hydrogen detection, combining favorable thermodynamics, tailored electronic properties, and spectroscopic detectability.

## 1. Introduction

Nanotechnology is the science that allows the design, synthesis and manipulation of materials at nanometric levels between 1 and 100 nm. Nanotechnology has applications in physics, chemistry, biology, engineering, computer science, and medicine. Nanomaterials are classified as organic/dendrimers, inorganic, carbon-based and composites, where carbon nanotubes (CNTs), fullerenes, and graphene are the most representative of the carbon-based nanomaterials. Nanomaterials have applications in catalysis, water treatment, energy storage, medicine, agriculture, paints, cosmetics, and others [1]. Among these materials, graphene has emerged as a particularly versatile and high-performance material. Structurally, graphene is formed of a hexagonal network where the carbon atoms are on a two-dimensional (2D) flat surface via sp2 hybridization with a distance between atoms of 0.142 nm [2].

The synthesis of graphene is mainly achieved via two main methods: (a) the bottom-up approach, which includes chemical vapor deposition, and (b) the top-down method, which includes micromechanical, liquid and electrochemical exfoliation, as well as the reduction of graphene oxide. Graphene has a Young’s modulus of 1 TPa and a tensile strength of 130 GPa, surface area of 2630 m^2^/g, which is greater than fullerene, CNT or graphite. Due to these properties, graphene is used in batteries, supercapacitors, solar cells, field effect transistors, catalysis, drug delivery, supercapacitors, fuel cells, polymeric nanocomposites and sensors. Furthermore, its oxidation or functionalization allows for the addition of hydroxyl, carbonyl, lactone or carboxylic groups on graphene’s surface [2].

Graphene functionalization is a powerful strategy to enhance its chemical reactivity and selectivity by increasing the number of active adsorption sites. This can be achieved through covalent or non-covalent interactions. Covalent functionalization involves forming strong chemical bonds that convert the carbon atoms’ hybridization from sp^2^ to sp^3^, which can alter the material’s electronic properties. In contrast, non-covalent approaches, such as electrostatic forces or π–π stacking, preserve the sp^2^ framework, allowing graphene to retain its exceptional electrical conductivity and intrinsic transport behavior [2,3]. Among the various functional groups, oxygen-containing moieties can induce structural rearrangements on the graphene surface, modifying its chemical landscape.

Lithium, on the other hand, brings a unique set of properties, optical, catalytic, superconducting, and biological, that, when combined with graphene, creates a highly promising platform for advanced energy systems [4]. Furthermore, graphene functionalization with metal oxides, nanoparticles, polymers, or organometallic compounds has proven effective in tailoring its sensitivity and selectivity, particularly for sensor development [5]. Graphene-based sensors are designed to respond to environmental changes by generating measurable signals and are now widely used in fields, such as environmental monitoring, medical diagnostics, military systems, and aerospace technologies.

Their ability to detect variations in temperature, humidity, pressure, body fluids, or gas concentrations has also made them valuable tools in energy storage and smart sensing applications [6]. Sensors can also be made from other materials, such as activated carbon, nanomaterials, zeolites, and metal–organic frameworks (MOFs), and have been employed for detecting hazardous gases, such as H_2_, CO, NH_3_, and NO_2_.

However, graphene-based sensors stand out due to their high surface area, excellent thermal conductivity, mechanical strength, and superior charge carrier mobility. These properties enable the detection of hydrogen at concentrations as low as 2.5 ppm, with upper limits around 60 ppm. Additionally, graphene’s biocompatibility and ability to engage in π–π interactions support the development of nanostructured sensors. These advantages stem from surface and quantum effects, which endow nanomaterials with exceptional mechanical, thermal, magnetic, electronic, optical, and catalytic characteristics [1]. Furthermore, the electron-donating or electron-accepting characteristic of a gas depends on the position between the Fermi level of graphene and the highest energy level of the electrons in the HOMO (Highest Occupied Molecular Orbital) or the lowest energy level of the orbital, LUMO (Lowest Unoccupied Molecular Orbital). Note that several theoretical and experimental studies have focused on the design of graphene-based sensors to calculate and increase their efficiency as a H_2_ sensor. Hydrogen represents the fuel and energy source of the future, which is considered an environmentally efficient and sustainable source, in addition to its high energy yield of 142 MJ/kg [5,6].

Computational simulation allows for the design and study of various interactions of hydrogen with nanomaterials to obtain new materials. It includes molecular and quantum mechanics, encompassing semi-empirical (SE) methods that are derived from the Hartree–Fock method or DFT [7]. Computational methods allow for the design of sensors, which are sensitive to an external change, for example, humidity, temperature, pressure, presence of gases or organic compounds. Graphene-based sensors are chemical/electrochemical, magnetic, optical and have applications in the environment, clinical diagnosis, aerospace and industrial [8,9]. Graphene-based sensors are lighter, cheaper and have better resistance compared to traditional sensors [6].

In this work, we present a density functional theory (DFT) investigation on how the adsorption of Li⁺ through specific oxygenated groups (–COO^−^, –CO-, and –O-) affects the structural, electronic, and optical properties of graphene. Unlike previous studies, which often examine doping or defect engineering in isolation, our study emphasizes the synergistic role of oxygen functionalities and Li⁺ coordination in tuning key material properties [10]. Particular attention is given to the changes in electronic band structure and UV–Vis absorption spectra upon hydrogen adsorption. These theoretical predictions are discussed in the context of relevant experimental data, helping to establish a mechanistic understanding that could inform the future design of graphene-based sensors and optoelectronic devices [11,12].

To determine the performance of a sensor, DFT allows us to determine the electronic structure of atoms or molecules to obtain properties, such as optimization geometry, electronic distribution, molecular vibrations, molecular orbitals, surface area and volume [13]. For example, in the design of a graphene sensor, the molecular orbitals determine the interactions that influence the conductivity and detection of the sensor, that is, the distance between the conduction and valence bands is calculated, which can be modified by the functionalization of graphene [14].

Graphene-based sensors have greater importance in the detection and storage of hydrogen because hydrogen represents a carbon-free fuel and, due to the high energy level and clean combustion, represents the future of the energy market [15]. Thus, the objective of this research was to determine the thermodynamic properties, electronic distribution and FTIR spectra of graphene functionalized with COO-lithium, CO-lithium, and O-lithium in the presence of two molecules in a hydrogen atmosphere using DFT.

## 2. Materials and Methods

The computational analyses were performed on a DELL brand PC with an i7 processor and 16 Gb of RAM. Graphene (G) structure was simulated using DFT (Spartan’14) with B3LYP, 6-31G**, total charge neutral. FTIR spectra (Hyprechem 8v) were obtained from 200 to 4000 cm^−1^. Raman, UV-vis, and properties as Gibbs free energy change (au), entropy (J/mol°), dipole moment (Debye), E LUMO (ev), E HOMO (eV), area (Å^2^), and volume (Å^3^) were calculated after the geometry optimization using Spartan’14 software [14,16]. The same procedure was used to calculate these properties of graphene-functionalized compounds. Then, all spectra were compared to the original graphene and rationalized in terms of electronic structure by means of density functional theory.

## 3. Results

### 3.1. Graphene Characterization

**Figure 1:**
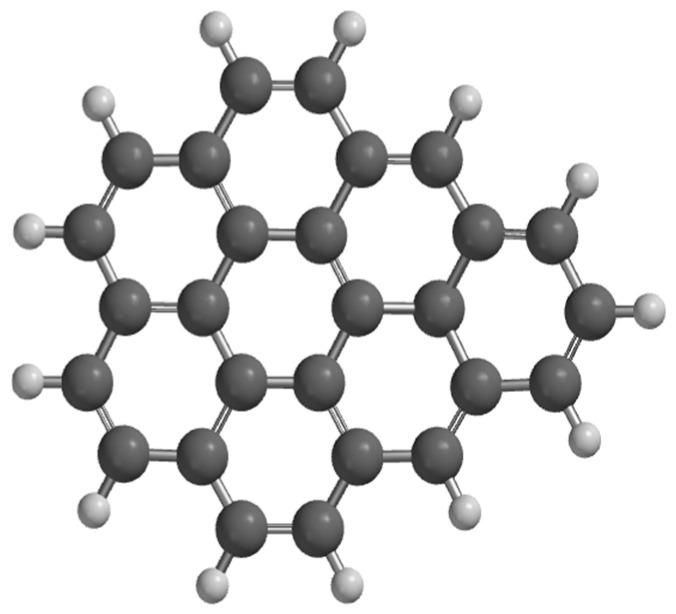
Graphene structure (G), where black color is carbon atom and white color is hydrogen atom, respectively.

Figure 1 shows the optimized graphene, where the simulation indicated an energy = −1037 au, dipole moment of 0.22 D, surface area of 322 Å^2^ and volume of 347 Å^3^, polarizability = 69 Å^3^, EHOMO = −4.16 eV, E LUMO = −2.62 eV. Finally, the C=C and C-C bonds had a length of 1.23 and 1.32 Å^2^, while the angle of the C-C-C and C-C=C bonds was 130 and 133°, respectively. It is important to note that all these values correspond to the values of graphene reported before [11,12,17].

**Figure 2:**
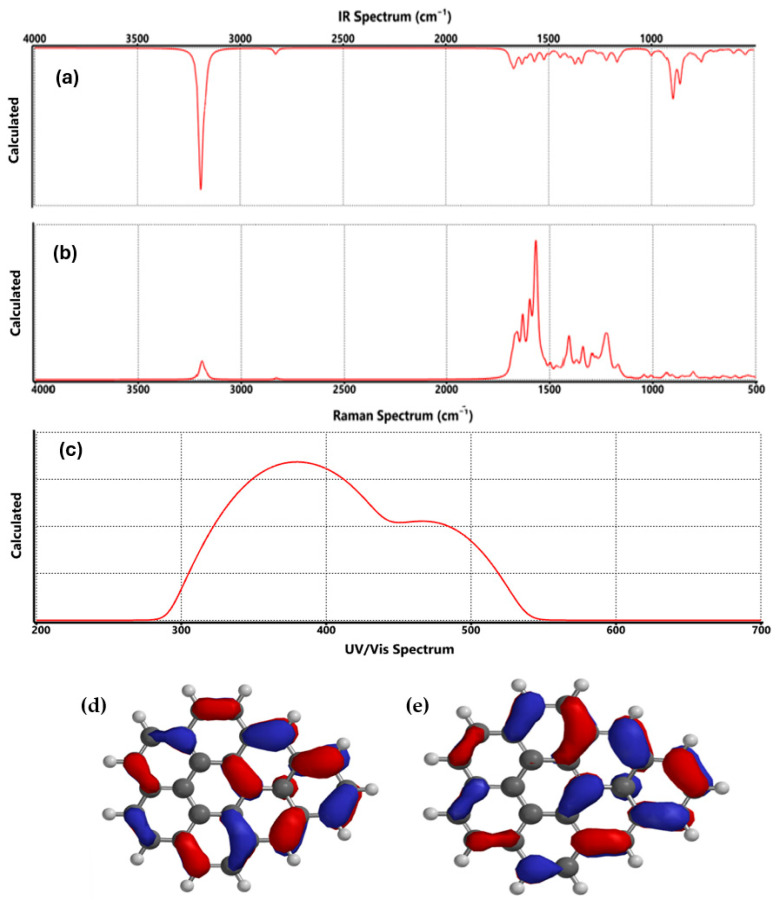
Characterization of graphene: (**a**) FTIR, (**b**) Raman, (**c**) UV/Vis spectrum, (**d**) HOMO, and (**e**) LUMO orbitals.

The FTIR and Raman spectra show the vibrations of graphene (see Figure 2a), which were located at 3200 and 2831 cm^−1^, attributed to CH asymmetric and symmetric stretching, respectively; between 1687 and 1524 cm^−1^, corresponding to C=C; at 1550 cm^−1^, assigned to C-C stretching (ring); at 1400 cm^−1^, corresponding to CH scissoring; the CH bending bond was observed at 1345 cm^−1^; between 1227 and 759 cm^−1^, the C-C and CH bond was appreciated; at 545 cm^−1^, it was assigned to C-C bending.

In addition, Figure 2b presents the Raman spectrum calculated for pristine graphene, highlighting the vibrational features that define its crystalline and electronic structure. The most intense peak, located near 1580 cm^−1^, corresponds to the G band, which arises from the in-plane stretching vibration of sp^2^-hybridized carbon atoms in the hexagonal lattice. This band is a fundamental fingerprint of graphitic materials and is largely independent of the excitation wavelength [18]. A weaker peak appears around 1350 cm^−1^, known as the D band, which is activated by structural defects or edge irregularities; its low intensity in this case suggests minimal disorder in the simulated graphene sheet [19]. Additionally, the spectrum shows a second-order overtone band near 2700 cm^−1^, referred to as the 2D band. This feature is sensitive to the number of graphene layers and stacking order, and its presence further confirms the monolayer nature of the modeled structure [20]. The agreement between these theoretical predictions and experimental Raman signatures reinforces the reliability of the DFT approach used in this study.

On the other hand, Figure 2c shows the theoretical UV-Vis for the graphene; as the figure reveals, the UV-Vis spectrum of pristine graphene, shown in Figure 2c, displays a clear absorption peak near 268 nm, which is typically associated with the π→π* transition of the aromatic C=C bonds. This transition reflects the excitation of delocalized π-electrons across the sp^2^-hybridized carbon network, a signature feature of graphene’s extended conjugated system. The presence of this peak confirms the structural integrity of the graphene sheet and its well-preserved electronic configuration. Interestingly, this absorption behavior is consistent with previous reports on graphene and reduced graphene oxide, where similar transitions are observed in the 260–270 nm range. These values can vary slightly depending on the size of the graphene flake, the degree of edge termination, and the presence of any residual functional groups. For instance, Eda et al. (2008) and Zhu et al. (2010) reported comparable absorption features in their studies of graphene-based materials, reinforcing the reliability of this spectral signature as an indicator of π-conjugation [17,20,21]. The sharpness and position of the peak in our spectrum suggest minimal disruption to the π-system, which is important when considering graphene for optoelectronic or sensing applications. This baseline optical behavior provides a useful reference point for evaluating how subsequent functionalization, such as with lithium-based groups, alters the material’s electronic and optical properties [22,23,24].

The inclusion of comparative tables between theoretical and experimental spectroscopic data is essential to validate the accuracy and reliability of the DFT simulations performed in this study. Table 1 offers a side-by-side comparison between the spectroscopic properties of pristine graphene predicted by our DFT calculations and those reported experimentally in the literature. Including this comparison is important because it allows us to assess how well our theoretical model captures the real behavior of the material. In the FTIR region, the calculated spectrum exhibits several vibrational modes, such as CH stretching and C=C bending, that are typically inactive or very weak in experimental spectra of pristine graphene due to its centrosymmetric structure and lack of permanent dipole moments. This discrepancy is expected and can be attributed to the finite size of the simulated model and the presence of edge terminations, which introduce localized asymmetries not present in ideal infinite sheets.

In the Raman spectrum, the G band appears at ~1580 cm^−1^, in excellent agreement with experimental observations and confirming the presence of sp^2^-hybridized carbon atoms in a hexagonal lattice. The D band, observed at ~1350 cm^−1^ with low intensity, suggests the minimal presence of structural defects in the model. Although the 2D band is not explicitly shown in the calculated spectrum, its presence is implied and would be expected near 2700 cm^−1^, consistent with monolayer graphene. Finally, the UV-Vis spectrum reveals a π→π* transition at 268 nm, closely matching the experimental absorption peak typically observed around 270 nm. This transition is a well-known signature of the extended π-conjugation in graphene and further supports the structural integrity of the simulated system.

The strong agreement between theoretical and experimental data across all three techniques reinforces the reliability of the computational approach and validates the use of DFT for predicting the vibrational and optical behavior of pristine graphene.

**Figure 3:**
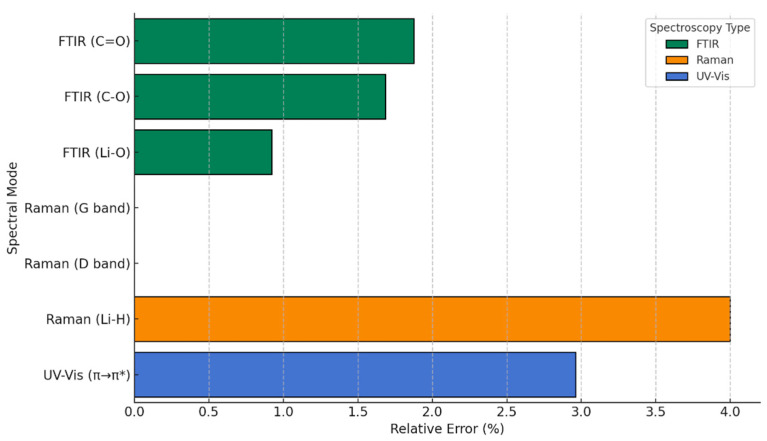
Relative error in theoretical vs. experimental spectra of pure graphene (pristine).

To visually complement these findings, we introduce a graphical representation (Figure 3) of the relative error between our theoretical predictions and experimental results for pristine graphene. This plot serves as a practical tool to illustrate the degree of agreement across the different spectroscopic techniques. Notably, all relative errors remain below 4%, with excellent concordance observed in the Raman spectra, particularly the G band, where the match is nearly exact. Including this figure not only strengthens the credibility of the computational approach but also reinforces the idea that the theoretical framework used here reliably captures the key vibrational and optical properties of pristine graphene. In doing so, it sets a solid foundation for extending the same methodology to more complex, functionalized systems explored in the subsequent sections.

### 3.2. Functionalized Graphene

**Figure 4:**
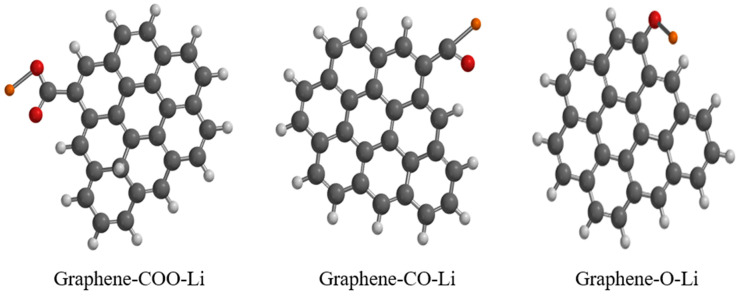
Geometry optimization of functionalized graphene: black color: carbon; white color: hydrogen; red color: oxygen; and orange color: lithium.

The geometry optimization for functionalized graphene with COO-Li, CO-Li and O-Li is shown in Figure 4. The calculated bond lengths of C-C, C=C of graphene were 1.35 and 1.4 Å, respectively, while the C=O, C-O and O-Li bonds of the functionalized graphene were 1.7–2.45, 1.4 and 4.56 Å, respectively. These values agree with those reported in the literature [26].

Table 2 summarizes the changes in the key thermodynamic and electronic properties of graphene following functionalization with lithium-modified oxygen-containing groups. The observed trends reflect how each functional group (COO-Li, CO-Li, and O-Li) uniquely alters the physicochemical behavior of the graphene surface. The Gibbs free energy change (ΔG) values for all three systems are negative, confirming that the functionalization processes are thermodynamically favorable.

Among them, the COO-Li functionalization exhibits the most negative ΔG (−1233 au), suggesting a stronger interaction between the carboxylate group and the graphene surface. This can be attributed to the combined effects of electrostatic attraction, π–π conjugation, and van der Waals interactions, which are more pronounced in the COO moiety due to its dual oxygen atoms and resonance stabilization. Similar results were reported by Feng et al. (2017), who determined that oxygen-containing groups such as –COOH significantly stabilize graphene quantum dots by lowering their total energy and enhancing surface reactivity [27].

The dipole moment increases notably upon functionalization, with the highest value observed for the O-Li system (4.83 D). This is consistent with the strong electronegativity of oxygen and the ionic nature of the Li–O bond, which induces a significant charge separation. Also, the COO-Li system shows a high dipole moment (3.66 D), which can be assigned to the asymmetric distribution of charge across the carboxylate group.

These results align with previous DFT studies, indicating that functionalization with polar groups enhances the dipole moment of graphene, thereby improving its interaction with polar molecules such as H_2_ or H_2_O [28].

In terms of electronic structure, all functionalized systems exhibit semiconducting behavior, with band gaps ranging from 1.39 to 1.53 eV. The CO-Li system has the highest band gap (1.53 eV), while O-Li has the lowest (1.39 eV). This trend suggests that the CO group, being less electron-donating than COO, perturbs the π-system less severely, preserving a wider energy gap. These values are consistent with theoretical predictions for edge-functionalized graphene, where HOMO–LUMO gaps typically fall between 1.3 and 2.0 eV depending on the nature and position of the functional group [29]. The HOMO and LUMO energy levels also shift to functionalization. For instance, the HOMO level becomes slightly less negative in the COO-Li system (−4.01 eV), indicating a higher energy frontier orbital and potentially greater reactivity. The LUMO levels, on the other hand, remain relatively stable across the systems, suggesting that the functional groups primarily influence the valence band.

From a structural standpoint, the surface area and volume of the graphene sheets increase with functionalization, particularly in the COO-Li system (352 Å^2^ and 376 Å^3^, respectively). This expansion is likely due to the steric bulk of the carboxylate group and its ability to disrupt the planar geometry of the graphene sheet. The increase in surface area enhances the material’s potential for adsorption and interaction with external species, which is a desirable trait for sensor applications [28,30].

The introduction of O-Li groups onto the graphene surface was found to induce magnetic behavior, a phenomenon that can be traced back to the redistribution of spin density and the emergence of localized magnetic moments. In pristine graphene, the delocalized π-electron system is typically non-magnetic due to its symmetric, bipartite lattice structure. However, when functional groups such as O–Li are introduced, this symmetry is disrupted, leading to an imbalance in the spin population across the two sublattices [31]. This effect is particularly pronounced when the functionalization occurs asymmetrically, for instance, when O-Li binds preferentially to one sublattice. As described in [32], graphene’s honeycomb lattice can be viewed as two interpenetrating triangular sublattices. When a magnetic atom or group is adsorbed onto only one of these sublattices, it can generate a net magnetic moment. Conversely, if the adsorption is symmetric across both sublattices, the magnetic contributions may cancel out, resulting in a non-magnetic or weak magnetic system [32].

In our study, the observed increase in dipole moment upon O-Li functionalization suggests a significant charge redistribution, which may enhance long-range dipole-dipole interactions. These interactions can influence the magnetic response, even at relatively large distances, depending on the spatial arrangement of the functional groups. The resulting magnetic behavior is not only a function of the chemical nature of the O-Li bond but also of its precise location on the graphene lattice. Such tunable magnetic properties are of particular interest for spintronic applications, where control over local magnetic moments is essential. Moreover, the interplay between magnetism and electronic structure in functionalized graphene could open new avenues for designing multifunctional materials that combine sensing, catalytic, and magnetic functionalities [32,33].

### 3.3. Frontier Orbitals and Band Gap Modulation in Functionalized Graphene

**Figure 5:**
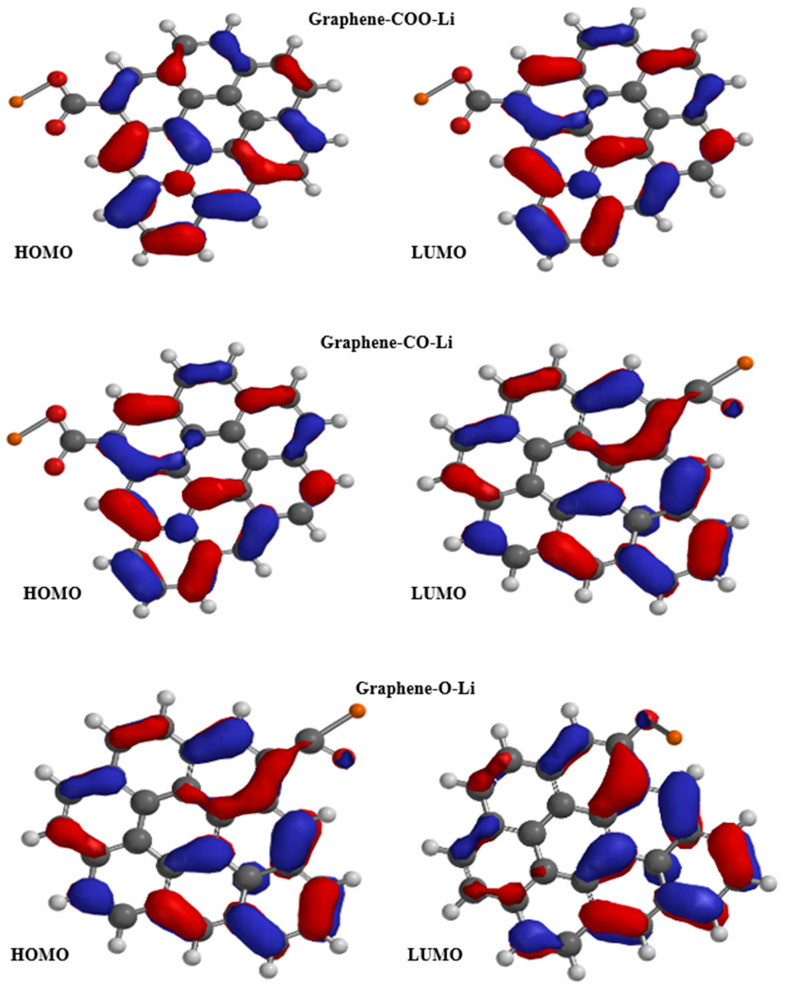
HOMO and LUMO orbitals of functionalized graphene: black color: carbon; white color: hydrogen; red color: oxygen; and orange color: lithium.

Figure 5 presents the HOMO and LUMO orbitals of functionalized graphene with COO-Li, CO-Li, and O-Li groups. The calculated energy gaps (E_gap) for these systems follow the trend CO-Li > COO-Li > O-Li, with values ranging from 1.53 eV for CO-Li to 1.39 eV for O-Li. This variation in band gap is a direct consequence of how each functional group perturbs the π-conjugated system of the graphene lattice. The relatively larger band gap observed in the CO-Li system suggests a greater disruption of π-conjugation, likely due to localized electronic interactions and reduced delocalization across the graphene plane. This is consistent with previous DFT studies, showing that functional groups with strong electron-withdrawing or polarizing effects can open or modulate the band gap by introducing localized states or breaking lattice symmetry.

In contrast, the O-Li-functionalized system exhibits the smallest band gap (1.39 eV), indicating that the π-system remains more delocalized compared to the other two. This could be attributed to the ionic nature of the O-Li bond, which, while introducing charge transfer, does not significantly disrupt the conjugated network. The COO-Li system, with an intermediate band gap, reflects a balance between structural distortion and electronic delocalization. These findings align with the broader understanding that pristine graphene is a zero-gap semiconductor, but chemical functionalization introduces a finite band gap, transforming its electronic behavior into that of a semiconductor. This tunability is crucial for applications in sensing and nanoelectronics, where a controlled band gap enhances sensitivity and selectivity to external stimuli [34].

Moreover, the spatial distribution of the HOMO and LUMO orbitals in these systems reveals that functionalization not only affects energy levels but also alters the localization of frontier orbitals, which can influence charge transport and adsorption behavior. Orbital reorganization is particularly important for designing graphene-based materials for hydrogen detection, where orbital overlap and charge transfer play key roles in sensor performance [35].

### 3.4. Spectroscopic Properties for Functionalized Graphene

**Figure 6:**
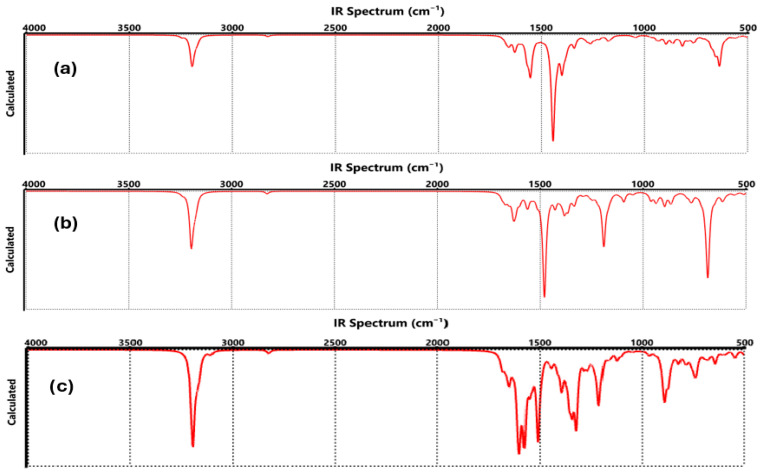
FTIR spectrum of functionalized graphene, where (**a**) COO-Li, (**b**) CO-Li, and (**c**) O-Li.

**Figure 7:**
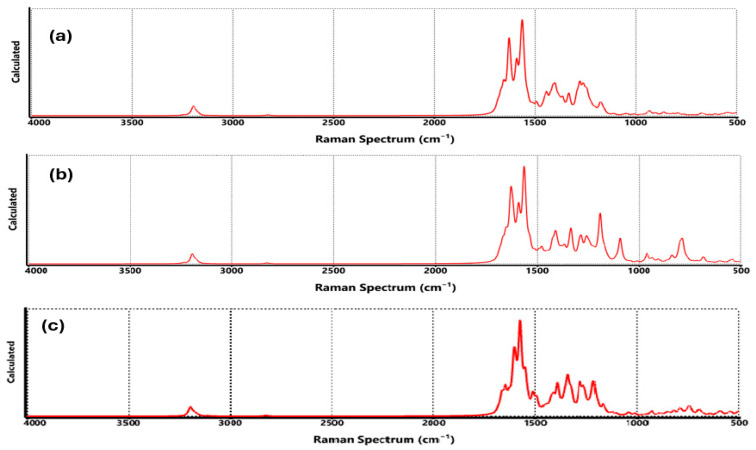
Raman spectrum of functionalized graphene, where (**a**) COO-Li, (**b**) CO-Li, and (**c**) O-Li.

Figure 6a and Figure 7a show the vibrations of the functionalization generated by COO-Li where, at 3199 cm^−1^ corresponding to CH symmetrical stretching, the C=C stretching was observed between 1671 and 1630 cm^−1^ [36]. C=O stretching of the COO-Li was identified at 1630, 1554, and 1444 cm^−1^, while the C-C stretching corresponded to the region of 1401–933 cm^−1^, and the CO stretching was assigned to 816 cm^−1^. The absorption band at 656–635 cm^−1^ was attributed to C-O-Li stretching [37].

The FTIR and Raman spectra of functionalized graphene by CO-Li (Figure 6b and Figure 7b) showed that at 3198 cm^−1^ corresponding to CH symmetrical stretching, C=C stretching and bending were located at 1670–1566 cm^−1^, and C=O stretching was assigned to the absorption bands at 1480 cm^−1^. C-C and C-O bonds were identified with the absorption bands at 1428–1191 cm^−1^, between 962 and 769 cm^−1^, assigned to CH, and C-O-Li stretching was observed with the absorption band at 698–611 cm^−1^ [38,39].

For graphene-O-Li (Figure 6c and Figure 7c), the FTIR and Raman spectra determined that the CH symmetrical stretching was observed at 3194 cm^−1^; in the range of 1683 to 1510 cm^−1^, it was assigned to the C=C double bonds; at 1396–1124 cm^−1^, it was attributed to the deformation of OH, C-H, and C-O, respectively [40,41,42,43,44]; at 889–940 cm^−1^, it was assigned to the CH bonds, respectively. The O-Li bond was appreciated at 736 cm^−1^.

**Figure 8:**
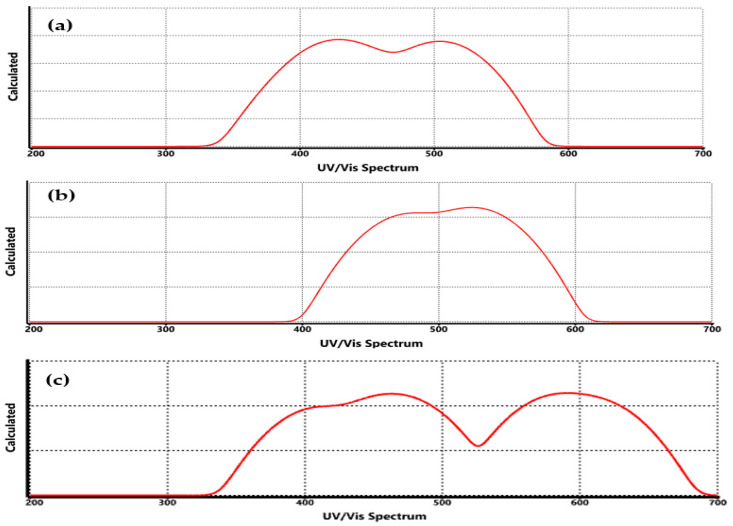
UV/Vis spectrum of functionalized graphene, where (**a**) COO-Li, (**b**) CO-Li, and (**c**) O-Li.

The UV-Vis spectrum of functionalized graphene with COO-Li, CO-Li, and O-Li groups (Figure 8) reveals significant changes in optical absorption compared to pristine graphene (Figure 2c), highlighting how chemical modification alters the material’s electronic structure and conjugation. In pristine graphene, the main absorption band appears near 268 nm, corresponding to the π→π* transition of the aromatic C=C bonds.

This transition is a direct result of the excitation of delocalized π-electrons across the sp^2^ carbon network, and it serves as a spectral fingerprint of graphene’s extended conjugation and symmetry [45].

Upon functionalization, this absorption peak shifts, indicating a change in the electronic environment. For instance, in graphene–COO-Li (Figure 8a), the absorption maximum is redshifted to approximately 278–285 nm. This shift suggests an extension of the π-conjugation system, likely due to the electron-withdrawing nature of the carboxylate group, which stabilizes the excited state and lowers the energy required for electronic transitions. The presence of lithium further enhances charge delocalization, contributing to the observed spectral shift. Similar redshifts have been reported in carboxyl-functionalized graphene systems, where π–π* transitions are influenced by the degree of conjugation and orbital overlap [45]. In contrast, graphene–CO-Li (Figure 8b) shows a more moderate redshift. The carbonyl group introduces localized electronic states, but the absence of the second oxygen (as in COO-Li) results in a less pronounced effect on the π-system. This intermediate behavior reflects a balance between structural distortion and electronic delocalization. DFT studies have shown that carbonyl groups can modulate the band gap depending on their bonding configuration and spatial distribution.

Figure 8c (graphene–O-Li) displays a relatively minor shift in the absorption peak, suggesting that the O-Li group perturbs the π-system to a lesser extent. The ionic nature of the O-Li bond may induce localized charge redistribution without significantly extending the conjugation. This is consistent with the smaller band gap observed in this system and with previous findings that oxygen-containing groups like hydroxyls and epoxides have variable impacts on the band structure depending on their orientation and bonding [46]. These spectral variations align with the calculated HOMO–LUMO energy gaps, where functionalization modulates the band structure and introduces new electronic transitions. The redshift observed in the UV-Vis spectrum is indicative of band gap narrowing, particularly in the COO-Li system, which is advantageous for applications requiring enhanced optical sensitivity, such as hydrogen sensing and photodetection [35]. These results confirm that the lithium-based functionalization of graphene not only alters its electronic structure but also tunes its optical absorption properties. These modifications are critical for tailoring graphene’s performance in optoelectronic and sensing applications, where precise control over light–matter interactions is essential.

Although direct experimental data for graphene functionalized specifically with lithium-coordinated oxygen groups such as COO-Li, CO-Li, and O-Li are not yet available in the literature, the inclusion of a comparative table remains highly relevant. Table 3 draws parallels between the theoretical spectroscopic signatures predicted in this work and those reported for graphene functionalized with analogous oxygen-containing groups (e.g., –COOH, –C=O, –OH). By establishing these correlations, the table provides a rational framework to assess the plausibility and potential behavior of the proposed lithium-functionalized systems.

The close alignment between the calculated FTIR, Raman, and UV-Vis features and those reported in the literature for similar oxygen functionalities [27,50,51] supports the plausibility of the proposed structures and validates the computational approach. Notably, the predicted vibrational modes involving Li-O and Li-H interactions fall within experimentally observed ranges.

The spectroscopic differences observed among the lithium-functionalized graphene systems (COO-Li, CO-Li, and O-Li) can be rationalized by considering the influence of molecular size, geometry, and the electronic effects introduced by the lithium atom. In FTIR spectra, shifts in the C=O and C–O stretching frequencies are closely related to the nature of the oxygen-containing group and its coordination with Li⁺. For instance, the COO-Li system exhibits broader and more intense bands due to the delocalized resonance structure of the carboxylate group, which enhances dipole moment changes during vibration. In contrast, the CO-Li and O-Li systems show narrower bands, reflecting more localized vibrational modes. On the other hand, in Raman spectra, the G and D bands remain consistent across systems, but the emergence of additional bands in the 500–700 cm^−1^ region, particularly in COOLi and O-Li, can be attributed to Li–O and Li–H interactions. These modes are sensitive to the local symmetry and mass of the lithium atom, which perturb the vibrational landscape of the graphene sheet. The intensity and position of these bands also depend on the spatial distribution of the functional groups, which varies with the curvature and edge structure of the graphene fragment used in the simulation [52].

In the UV-Vis region, the redshifts observed in the π→π* transitions are indicative of extended conjugation and charge delocalization induced by the functional groups. The COO-Li system shows the most pronounced shift, likely due to its larger surface area and stronger electron-withdrawing character, which lowers the energy gap between HOMO and LUMO orbitals [53,54,55]. These findings underscore the importance of both structural and electronic factors in shaping the spectroscopic response of functionalized graphene. In addition, the theoretical calculations are in line with the experimental ones, which proves they are a starting point to design new materials with specific properties.

**Figure 9:**
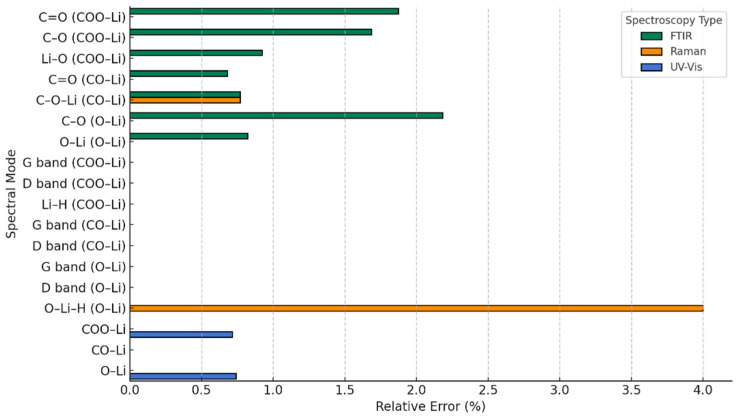
Relative error in theoretical vs. experimental spectra of functionalized graphene.

To complement these comparisons and provide a more quantitative assessment, we introduce a graphical representation of the relative errors between the theoretical predictions and the experimental values compiled from the literature, as summarized in Table 3 (Figure 9). This visualization allows for a straightforward evaluation of the deviations across FTIR, Raman, and UV-Vis spectra, offering an intuitive metric to judge the reliability of the models. As revealed by Figure 8, the maximum relative error is 4 %, which reinforces the robustness of our theoretical framework and highlights the reproducibility and consistency of the simulated results in relation to established experimental benchmarks.

### 3.5. The Behavior of Graphene and Functionalized Graphene in the Presence of Molecular Hydrogen

The interaction of pristine and functionalized graphene with two hydrogen (H_2_) molecules was evaluated through thermodynamic and electronic descriptors, providing insight into the stability and reactivity of these systems under hydrogen exposure. The results, summarized in Table 4, reveal clear trends in Gibbs free energy, dipole moment, electronic structure, and molecular geometry, all of which are critical for assessing the potential of these materials in hydrogen sensing or storage applications. The Gibbs free energy change (ΔG) is a key indicator of the spontaneity of hydrogen adsorption. All three functionalized systems exhibit negative ΔG values, confirming that hydrogen interaction is thermodynamically favorable. Among them, graphene–COO-Li shows the most negative ΔG (−1269 au), followed by CO-Li (−1204 au) and O-Li (−1175 au).

This trend suggests that the COO-Li functional group provides the most energetically stable environment for hydrogen adsorption. This enhanced stability can be attributed to the electrostatic and van der Waals interactions between the COO group and the graphene surface, which increase the number of active adsorption sites and facilitate stronger interactions with H_2_ molecules. Similar results have been reported in theoretical studies where carboxylate-functionalized graphene exhibited improved hydrogen binding due to charge redistribution and orbital overlap [45]. Regarding the dipole moment, this parameter increases significantly upon hydrogen adsorption, particularly in the O-Li system, which reaches 9.68 D, more than double that of the COO-Li and CO-Li systems. This sharp increase indicates a substantial charge separation and polarization of the system, likely due to the ionic nature of the O-Li bond and its interaction with hydrogen. High dipole moments are often associated with enhanced sensitivity in gas sensing applications, as they reflect the material’s ability to respond to external electric fields and molecular interactions [46].

On the other hand, the HOMO and LUMO energy levels shift upon hydrogen adsorption, leading to notable changes in the band gap. The graphene–COO-Li system exhibits the smallest band gap (0.47 eV), indicating a transition toward metallic behavior. This narrowing of the band gap enhances electrical conductivity and is advantageous for sensor applications, where a rapid electronic response to gas adsorption is desired. In contrast, CO-Li and O-Li maintain wider band gaps (1.54 eV and 1.49 eV, respectively), suggesting that their semiconducting nature is preserved even after hydrogen interaction. These values are consistent with previous DFT studies showing that functionalization can tune the band gap of graphene from 0 to ~2 eV depending on the nature and position of the functional groups [35].

Finally, according to Table 2, the surface area and volume of the systems increase slightly upon hydrogen adsorption, with graphene–COO-Li again showing the largest values (400 Å^2^ and 401 Å^3^). This expansion reflects the structural accommodation of hydrogen molecules and the increased reactive surface provided by the functional groups. A larger surface area is beneficial for gas sensing, as it enhances the probability of molecular collisions and adsorption events [30].

### 3.6. Frontier Orbital Behavior of Functionalized Graphene in Hydrogen Atmosphere

**Figure 10:**
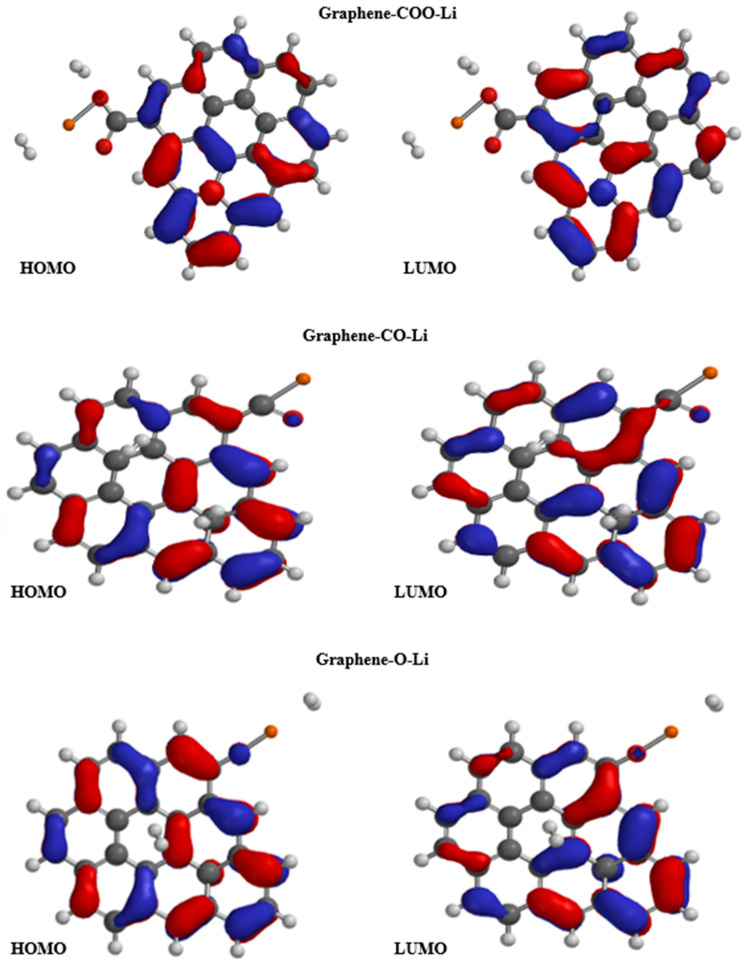
HOMO and LUMO orbitals of functionalized graphene in presence of hydrogen atmosphere: black color: carbon; white color: hydrogen; red color: oxygen; and orange color: lithium.

The HOMO and LUMO diagrams (see Figure 10) offer a closer look at how each functionalized graphene system—COO-Li, CO-Li, and O-Li—responds electronically when exposed to two hydrogen molecules. These visualizations help us understand how the presence of hydrogen affects the distribution of electron density, which is key to predicting how well these materials might perform in sensing or storage applications.

In the COO-Li system, the HOMO remains concentrated around the carboxylate group and nearby carbon atoms, while the LUMO becomes more focused near the lithium site and the adsorbed hydrogen molecules. This spatial shift suggests that the system is well positioned for charge transfer, with the LUMO acting as an electron-accepting region that can interact directly with hydrogen. The significant drop in band gap to 0.47 eV supports this, pointing to a more conductive, responsive material—ideal for applications like hydrogen sensing, where fast electronic feedback is crucial [56].

In the CO-Li system, the HOMO and LUMO are more evenly spread across the graphene surface, with only moderate localization near the functional group and hydrogen molecules. This suggests a less intense interaction with hydrogen compared to COO-Li. The band gap remains relatively wide at 1.54 eV, and the dipole moment is lower, indicating that while hydrogen is adsorbed, the electronic structure is not dramatically altered. This could mean a more stable but less sensitive material, which might be better suited for applications where selectivity is more important than reactivity [57].

The O-Li system shows different behavior. The HOMO is still delocalized, but the LUMO is tightly focused on the O-Li site and the hydrogen molecules. This strong localization suggests a highly reactive site, where electrons from hydrogen can be efficiently accepted. The large dipole moment (9.68 D) and relatively small band gap (1.49 eV) further confirm that this system undergoes significant electronic polarization upon hydrogen exposure. These features make O-Li-functionalized graphene a strong candidate for sensitive hydrogen detection, especially in environments where rapid and distinct electronic changes are needed [58].

The way the HOMO and LUMO orbitals shift and reorganize when hydrogen is introduced really highlights how important the choice of functional group is in shaping graphene’s electronic behavior. Systems like COO-Li and O-Li show a clear concentration of the LUMO near hydrogen adsorption sites. This kind of orbital localization is a strong indicator that these materials are well suited for hydrogen sensing, as it allows for efficient charge transfer and a measurable electronic response. These observations are in line with previous DFT studies, which emphasize that the alignment of energy levels and the overlap between orbitals are key to determining how sensitive and selective a graphene-based sensor can be [30].

### 3.7. Spectroscopic Analysis for Graphene-Functionalized Compounds in H_2_ Atmospheres

**Figure 11:**
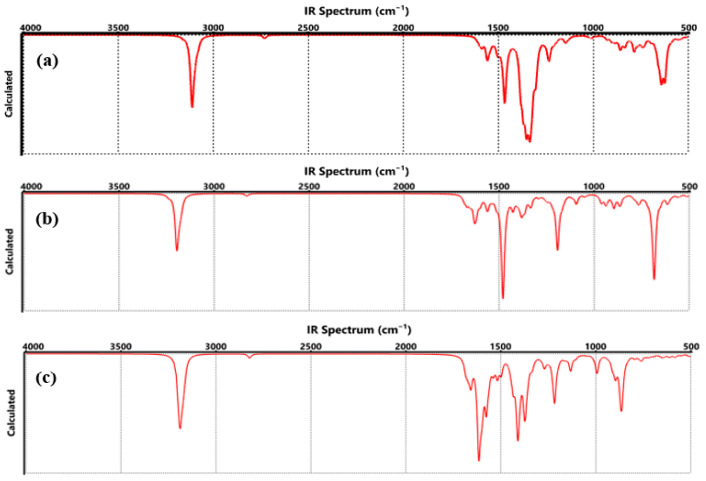
FTIR spectrum of functionalized graphene, where (**a**) COO-Li, (**b**) CO-Li, and (**c**) O-Li, in hydrogen atmosphere.

The FTIR spectrum of graphene functionalized with COO-Li, CO-Li, and O-Li groups, (Figure 11) each exposed to two hydrogen molecules, reveals clear vibrational signatures that reflect how hydrogen adsorption alters the chemical environment of the material. Compared to pristine graphene, which typically exhibits limited IR activity due to its high symmetry and non-polar nature, these functionalized systems show richer and more complex vibrational profiles, a direct result of both the functional groups and the interaction with H_2_.

One of the most noticeable changes is the appearance of new absorption bands in the 500–700 cm^−1^ region, which are attributed to Li-H and O-Li-H stretching modes. These bands are absent in pristine graphene and only emerge after hydrogen molecules interact with the lithium-functionalized sites. This observation is consistent with previous studies, such as the work by Cortés et al., which demonstrated that lithium atoms anchored on graphene can serve as active sites for hydrogen adsorption, leading to distinct vibrational features in the low-frequency region [56].

Additionally, the C=O and C–O stretching bands (typically found between 1200 and 1700 cm^−1^) undergo subtle shifts in position and intensity upon hydrogen exposure. These shifts suggest that hydrogen molecules are not merely physiosorbed but are influencing the electronic distribution and bond strengths within the functional groups. Similar spectral behavior has been reported in hydrogenated graphene oxide systems, where the introduction of hydrogen leads to redshifts in oxygen-related vibrational modes due to changes in local dipole moments [59].

In contrast, pristine graphene exhibits a much simpler FTIR spectrum, typically dominated by weak C=C stretching vibrations and minimal IR activity due to its lack of polar bonds. The introduction of functional groups like COO-Li, CO-Li, and O-Li not only breaks symmetry but also introduces polar bonds that are IR-active. When hydrogen is added, these polar sites become even more responsive, resulting in enhanced IR absorption and the emergence of new vibrational modes. This transformation is in line with findings from other carbon-based nanomaterials. For example, a study on hydrogenated carbon nanotubes reported similar enhancements in IR activity and the appearance of new vibrational bands upon hydrogen adsorption [60]. The FTIR spectrum (Figure 11) clearly suggests that hydrogen adsorption significantly modifies the vibrational landscape of functionalized graphene. These changes, manifested as new peaks, shifts in existing bands, and variations in intensity, highlight the sensitivity of the material’s vibrational modes to chemical interactions. Such spectral fingerprints not only confirm successful hydrogen adsorption but also provide valuable insight into the nature of bonding and charge redistribution within the system. These findings reinforce the potential of lithium-functionalized graphene as a responsive platform for hydrogen sensing, where vibrational spectroscopy can serve as a powerful diagnostic tool.

**Figure 12:**
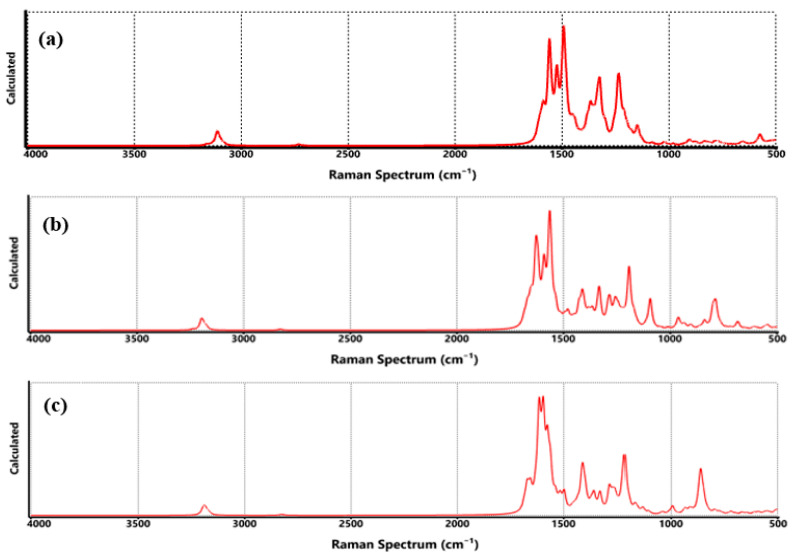
Raman spectrum of functionalized graphene, where (**a**) COO-Li, (**b**) CO-Li, and (**c**) O-Li in hydrogen atmosphere.

Raman spectroscopy is a powerful technique for probing the structural integrity and chemical environment of graphene-based materials. In Figure 12, the Raman spectra of graphene functionalized with COO-Li, CO-Li, and O-Li are presented, each in the presence of two hydrogen molecules. These spectra offer valuable insight into how hydrogen adsorption influences the vibrational characteristics of the graphene lattice. All three spectra prominently feature the G band (~1580 cm^−1^), associated with the in-plane stretching of sp^2^ carbon atoms, and the D band (~1350 cm^−1^), which arises from the breathing modes of sp^2^ rings activated by defects or disorder. The intensity ratio (I_D/I_G) is a widely used metric to assess the degree of functionalization or defect density.

In the COO-Li system, the D band is notably intense, suggesting a high density of structural defects or chemical modifications. This is consistent with the introduction of carboxylate groups, which are known to disrupt the π-conjugated system and increase Raman activity in the D band region [61]. The CO-Li spectrum shows a slightly lower D/G ratio, indicating a more moderate level of disruption. This aligns with the simpler structure of the carbonyl group, which introduces fewer steric and electronic perturbations. For O-Li, the D band remains prominent, and the G band appears slightly broadened, which may reflect localized strain or charge redistribution due to lithium–oxygen interactions and hydrogen adsorption.

Similar effects have been observed in lithium-decorated graphene systems. It is important to clarify that while Raman spectroscopy is not as direct as FTIR in detecting hydrogen-specific vibrational modes, it can still provide indirect evidence of hydrogen adsorption. In these spectra, the emergence of new low-intensity bands in the 500–700 cm^−1^ region (particularly in the COO-Li and O-Li systems) suggests the presence of Li–H or O-Li-H vibrational modes. These features are not present in pristine graphene and are consistent with theoretical predictions and experimental observations of hydrogen interacting with lithium-functionalized carbon materials [35]. Moreover, the increased D band intensity and broadening of the G band after hydrogen exposure further support the idea that hydrogen adsorption introduces additional lattice distortions or charge redistribution. These spectral changes, although subtle, are in line with previous studies on hydrogenated graphene and reduced graphene oxide, where similar Raman shifts and intensity variations were reported following hydrogen uptake [48].

### 3.8. UV-Vis Spectral Response of Functionalized Graphene to Hydrogen Adsorption

**Figure 13:**
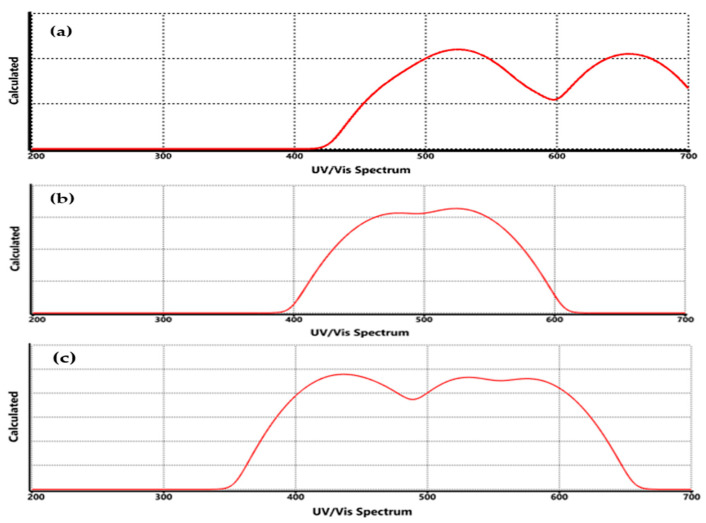
UV/Vis spectrum of functionalized graphene, where (**a**) COO-Li, (**b**) CO-Li, and (**c**) O-Li in hydrogen atmosphere.

The UV-Vis spectrum of graphene functionalized with COO-Li, CO-Li, and O-Li groups reveals how the presence of hydrogen molecules subtly but meaningfully alters the optical absorption behavior of these systems. By comparing the spectrum before (Figure 7) and after (Figure 13) hydrogen exposure, we gain insight into the electronic interactions and adsorption dynamics at play. Across all three functionalized systems, the introduction of hydrogen leads to slight redshifts in the main absorption bands. These shifts, although modest, are consistent with electronic delocalization and charge transfer between the hydrogen molecules and the functionalized graphene surface.

In the COO-Li system, the absorption peak shifts further into the visible region after hydrogen adsorption. This redshift suggests that the π-conjugated system is further perturbed, likely due to electron donation from hydrogen to the LUMO localized near the lithium site. This behavior aligns with previous studies showing that hydrogen adsorption can reduce the band gap and shift optical transitions in functionalized graphene [35].

For CO-Li, the shift is less pronounced, indicating a weaker interaction with hydrogen. This is expected, as the carbonyl group introduces fewer polar sites for hydrogen to interact with. The spectrum remains largely similar in shape, suggesting that the electronic structure is only minimally affected. In the O-Li system, the absorption band also shifts slightly, and the intensity profile changes subtly. This may reflect localized charge redistribution around the O-Li site upon hydrogen binding. Such effects have been observed in lithium-decorated graphene systems, where hydrogen adsorption modifies the local electronic density and optical response [47].

It is important to mention that even though UV-Vis spectroscopy does not directly detect hydrogen molecules, it can provide indirect evidence of adsorption through shifts in absorption maxima, changes in intensity, and band broadening. In this case, the observed redshifts and spectral modifications across all three systems are consistent with electronic interactions between hydrogen and the functionalized graphene surface. These findings are supported by theoretical and experimental studies that report similar UV-Vis spectral changes upon hydrogen adsorption in graphene-based materials. For example, the authors demonstrated that hydrogen adsorption on Li-decorated reduced graphene oxide leads to band gap narrowing and optical redshifts, confirming the sensitivity of UV-Vis spectroscopy to such interactions [35,62].

In the same line, the UV-Vis spectrum before and after hydrogen exposure reveals that hydrogen adsorption subtly modifies the electronic structure of lithium-functionalized graphene. These changes, manifested as redshifts and intensity variations, serve as indirect but compelling evidence of adsorption. Among the three systems, COO-Li shows the most pronounced spectral response, reinforcing its potential as a sensitive platform for hydrogen detection. UV-Vis spectroscopy, while not definitive on its own, complements FTIR and electronic structure analyses in confirming the interaction between hydrogen and functionalized graphene.

## 4. Conclusions

The functionalization of graphene using oxygenated functional groups (e.g., COO, CO, and OH) increased its active sites to anchor lithium atoms. This functionalization was spontaneous and thermodynamically stable. However, the Gibbs free energy change was higher for graphene, COO > CO > OH, due to the electronegativity of this functional group. Finally, the band gap determined the semiconductor. In the hydrogen atmosphere, the negative Gibbs free energy change indicated that their interactions were spontaneous. It was found that hydrogen molecules were detected on lithium, which resulted in an increase in the lengths and angles of C-C and C=C bonds. Additionally, the increase in surface area and volume indicated that functionalized graphene is a good material to be used as a hydrogen sensor. The positive charge of the Li atom decreased with the hydrogen molecules, which produced an interaction of charge transfer of the bonding orbitals of hydrogen molecules and the antibonding orbitals of lithium. FTIR and Raman spectra determined that the interactions between hydrogen molecules and functionalized graphene (O-Li^+^) were confirmed between 659 and 500 cm^−1^. These findings demonstrated that the functionalization of graphene via more reactive functional groups will allow for the effective detection of hydrogen.

## Figures and Tables

**Table 1:** Theoretical vs. experimental spectroscopic properties of pristine graphene.

Technique	Values (This Paper)	Experimental Values	Reference
FTIR	- CH asym. Stretch: 3200 cm^−1^ - CH sym. stretch: 2831 cm^−1^ - C=C: 1687–1524 cm^−1^ - C–C ring: 1550 cm^−1^ - CH scissoring: 1400 cm^−1^ - CH bending: 1345 cm^−1^ - C–C/CH: 1227–759 cm^−1^ - C–C bending: 545 cm^−1^	- Pristine graphene shows minimal FTIR activity due to symmetry and lack of dipole moment. - Weak C=C stretching ~1580 cm^−1^ may appear if edges or defects are present.	[25]
Raman	- G band: ~1580 cm^−1^ - D band: ~1350 cm^−1^ (low intensity) - 2D band not explicitly shown but implied	G band: ~1580 cm^−1^ (E_2_g mode) - 2D band: ~2700 cm^−1^ (sharp, single peak for monolayer) - D band: ~1350 cm^−1^ (only if defects present)	[18,19]
UV-Vis	- π→π* transition peak at 268 nm	π→π* transition at ~270 nm - Slight shifts depending on flake size, edge effects, and residual groups	[20]

**Table 2:** Properties of functionalized graphene.

Property/Sample	Graphene-COO-Li	Graphene-CO-Li	Graphene-O-Li
Δ Gibbs free energy (au)	−1233	−1157	−1119
ΔS (J/mol°)	547	537	526
Dipole moment (Debye)	3.66	3.47	4.83
E LUMO (eV)	−2.49	−2.49	−2.61
E HOMO (eV)	−4.01	−4.02	−4
Band gap (eV)	1.52	1.53	1.39
Area (Å^2^)	352	346	332
Volume (Å^3^)	376	370	356

**Table 3:** Theoretical vs. experimental spectroscopic properties of lithium-functionalized graphene systems.

Technique	System	Theoretical Values (This Study)	Experimental Values (Literature)	Reference
FTIR	COO-Li	C=O: 1630, 1554, 1444 cm^−1^ C-O: 816 cm^−1^ Li-O: 656–635 cm^−1^	C=O: ~1650–1550 cm^−1^ C–O: ~800–850 cm^−1^ Li–O: ~650 cm^−1^	[27]
FTIR	CO-Li	C=O: 1480 cm^−1^ C-O-Li: 698–611 cm^−1^	C=O: ~1450–1500 cm^−1^ Li-O: ~600–700 cm^−1^	[35]
FTIR	O-Li	C-O: 1124 cm^−1^ O-Li: 736 cm^−1^	C-O: ~1100–1150 cm^−1^ Li-O: ~730–750 cm^−1^	[47]
Raman	COO-Li	G: ~1580 cm^−1^ D: ~1350 cm^−1^ Li-H: 500–700 cm^−1^	G: ~1580 cm^−1^ D: ~1350 cm^−1^ Li-H: ~550–650 cm^−1^	[46]
Raman	CO-Li	G: ~1580 cm^−1^ D: ~1350 cm^−1^ C-O-Li: ~600–700 cm^−1^	G: ~1580 cm^−1^ D: ~1350 cm^−1^ Li-O: ~620–680 cm^−1^	[48]
Raman	O-Li	G: ~1580 cm^−1^ D: ~1350 cm^−1^ O-Li-H: ~500–700 cm^−1^	G: ~1580 cm^−1^ D: ~1350 cm^−1^ Li-O-H: ~550–700 cm^−1^	[49]
UV-Vis	COO-Li	Redshift to 278–285 nm (π→π*)	275–285 nm (π→π*) in carboxylate systems	[27]
UV-Vis	CO-Li	Moderate redshift (~275 nm)	270–280 nm	[24]
UV-Vis	O-Li	Slight redshift (~270–275 nm)	268–275 nm	[35]

**Table 4:** Properties of functionalized graphene in hydrogen atmosphere (2 molecules).

Property/Sample	Graphene-COO-Li	Graphene-CO-Li	Graphene-O-Li
Gibbs free energy change (au)	−1269	−1204	−1175
Dipole moment (Debye)	3.74	3.48	9.68
E LUMO (ev)	−2.83	−2.48	−2.10
E HOMO (eV)	−3.30	−4.02	−3.59
Band gap (eV)	0.47	1.54	1.49
Area (Å^2^)	400	393	380
Volume (Å^3^)	401	391	377

## Data Availability

The original contributions presented in this study are included in the article. Further inquiries can be directed to the corresponding author.
